# Virtual post-COVID-19 clinics in Saudi Arabia: navigating the effect of the pandemic with a national project

**DOI:** 10.3389/fpubh.2025.1460324

**Published:** 2025-07-30

**Authors:** Faisal Alenezi, Aeshah Alsagheir, Samar Amer, Lamyia Alzubaidi, Abdul-Aziz S. Alhomod, Tareef Alamaa

**Affiliations:** ^1^Deputy Minister Assistant for Hospitals, Services, Ministry of Health, Riyadh, Saudi Arabia; ^2^Family and Home Health Care Consultant, Assistant Agency for Hospitals Services, Ministry of Health, Riyadh, Saudi Arabia; ^3^Department of Public Health and Community Medicine, Zagazig University, Zagazig, Egypt; ^4^Assistant Agency of Public Health, Ministry of Health, Riyadh, Saudi Arabia; ^5^Consultant Diabetologist, Director of Technical Affairs and Ada’a in the Deputy for Hospitals Services, Ministry of Health, Riyadh, Saudi Arabia; ^6^Department of Emergency Medicine, King Fahad City, Riyadh, Saudi Arabia; ^7^Innovation and Regulatory Sandbox, Seha Virtual Hospital, Riyadh, Saudi Arabia; ^8^Deputy Minister of Curative Services, Ministry of Health, Riyadh, Saudi Arabia

**Keywords:** post-COVID-19 conditions, virtual clinics, digital transformation, Saudi Arabia, assessment, COVID-19, medical appointments, SARS-CoV-2

## Abstract

**Background:**

During the COVID-19 pandemic, digital health transformation in healthcare services has undergone significant changes, especially in Saudi Arabia (SA), which was one of the first countries not only to battle the COVID-19 pandemic but also extended to post-COVID-19 conditions (PCCs) through a national project to provide a virtual assessment to COVID-19 patients at least 4 weeks after infection. Therefore, we conducted this study from 16 February to 16 June 2022 in SA to determine the frequency of PCCs, provide the necessary care, and identify the risk factors that delayed their return to their pre-COVID-19 health status.

**Methods:**

A national project targeted all the registered 12,125 COVID-19 patients in the national register system by family physicians in the PCCs virtual clinics in the Medical Consultation Call Centre (937), using a validated assessment tool.

**Results:**

A total of 12,125 recovered COVID-19 patients were called and asked to complete a virtual assessment; 5,451 (45.1%) did not answer, and 5,913 (48.8%) agreed and finished the test; 4,973, or 84.2% of participants, did not report any PCCs. The most frequent PCCs were fatigue (201, 3.4%), coughing (246, 4.2%), dyspnea (209, 3.6%), loss of appetite or weight loss (43, 7.3%), and poor concentration (50, 8.4%). All they needed was assurance and information about health. A mere 384 (6.5%) needed to be referred to PHCCs. A number of factors were associated with the need for a referral, and the severity of the SARS-CoV-2 infection, age group, sex, vaccination status, and body mass index were significant predictors of returning to the pre-infection health status.

**Conclusion:**

In SA, the response rate to the virtual post-COVID-19 clinics was low, and no-show was the main limitation. PCCs are a prevalent condition that requires further investigation. Many factors can predict the return of participants’ pre-COVID-19 health status and participants’ referral to post-COVID-19 clinics.

## 1 Introduction

Saudi Arabia (SA) was one of the first nations to implement early and unprecedented preventative measures not only to fight against the coronavirus disease (COVID-19) but also to treat post-COVID-19 conditions (PCCs). By 16 February 2022, the World Health Organization (WHO) reported that the number of recovered COVID-19 cases was more than 128 million worldwide, 4,326,827 in SA (1). Currently, the precise definition of PCCs is problematic, as there are no standardized definitions or guidelines. Moreover, the epidemiological data of PCCs vary across countries due to many factors, including the accuracy of self-reporting symptoms, the diagnosis, the length of the follow-up period, differences in healthcare system capability, and the reporting system (2).

PCCs are a multisystem disease characterized by a wide range of new, recurring, or ongoing health conditions. They have various types and combinations of health conditions for varying durations, ranging from mild to incapacitating, and even asymptomatic people can have PCCs (3). PCCs should be managed from a holistic perspective according to a comprehensive, multidisciplinary plan based on their patients’ presenting symptoms, underlying medical and mental disorders, and personal and social circumstances. Therefore, the workforce in public health—politicians, scientists, healthcare services, and the remainder of society—must collaborate (4, 5).

Globally, COVID-19 is a significant burden on healthcare systems, and healthcare services have encountered fundamental changes. The recovered patients’ needs for healthcare increased, resulting in the management of COVID-19 cases. The pandemic’s indirect effects on patients, particularly those with chronic medical conditions and PCC concerns, increased. Therefore, telemedicine makes it easier for individuals to get the required healthcare services while staying in their own homes, with lower costs, making the service more flexible, and reducing risks that have more positive effects on patients’ satisfaction (6).

The Saudi Ministry of Health (SMOH) launched the Kingdom’s first virtual hospital as part of ongoing efforts to digitalize the healthcare sector and “remote clinics” medical service on 26 January 2021, which enables patients to consult with a physician online via the Anat App (a free App to facilitate communication between medical professionals, make their jobs easier, improve the caliber of medical care that patients receive, allow users to view 2020 medical events, and e-prescribe prescriptions), Sehaty apps (a unified platform of MOH, which allows users to get several health services and access health information), and the telehealth clinic (THC) to achieve the third objective of the 15 objectives of the Saudi National Transformation Programme (NTP) 2030 (7, 8). The SMOH’s strategy involves promoting the use of telemedicine in Saudi Arabia, aiming to enhance the effectiveness and efficiency of healthcare. This includes slowing the spread of COVID-19 in medical facilities, facilitating faster and more convenient access to medical advice and services for non-critical patients, and monitoring and early detection of PCCs, thereby reducing the need for in-person visits. Information technology and digital transformation enable this (9, 10).

On 8 June 2022, the Minister of Hospital Services launched the national post-COVID-19 Clinical Services across all 20 health regions in the SA, providing comprehensive, continuous, evidence-based clinical care to all COVID-19 cases in the SA with a rationalized use of resources. The goal is to detect PCCs early and provide effective management services to all citizens and residents, thereby reducing the impact of the disease on the health services of infected individuals, improving their overall health status, and optimizing their function and quality of life. The focus is on advancing understanding of PCCs through the three “Rs” of recognition, research, and rehabilitation, in response to the World Health Organization and the global initiatives launched on 17 August 2022 (8–12).

There are relatively few volumes of published literature describing PCCS and its management plans. Therefore, we conducted this national project using digital transformation technology in SA Anat, Ma’uad, and virtual clinics at the medical consultation center with the following objectives: We conducted the study among COVID-19 patients at least 4 weeks after the onset of infection, from 16 February to 16 June 2022, in SA. Our objectives were to determine the frequency of PCCs, provide the necessary management, and explore the predictors of participants’ return to their pre-COVID-19 health status and the predictors of participants’ referral to post-COVID-19 clinics.

## 2 Methodology

### 2.1 Study design and setting

An interventional study was conducted in post-COVID-19 clinics at the Medical Consultation 937 call center from 16 February to 16 June 2022, in SA.

### 2.2 Study population

This study targeted all *confirmed positives for the* SARS-CoV-2 virus according to the Saudi Ministry of Health Protocol (SMOP) (13). Saudi and non-Saudi, both sexes, all age groups, and those who were asymptomatic or mildly to moderately ill with COVID-19 were all enrolled in the Health Electronic Surveillance Network (HESN) national registry system, and no cases were excluded.

PCR was used to diagnose the SARS-CoV-2 virus, which, in accordance with WHO guidelines, was measured by having competent, well-trained medical specialists take nasopharyngeal swabs from the patient’s left and right nasal cavities. The sample was then stored in a sample collection tube with 3 mL of regular viral transport medium inside (14).

### 2.3 The study (national project) phases

#### 2.3.1 *The first phase:* the preparation of the assessment (data collection) tool

It was prepared based on earlier studies (12, 15–22). It was first prepared in English before being translated into Arabic. The Arabic version of the English questionnaire was translated by a bilingual team of two medical experts and one externally certified medical translator. The back translation was completed by two English-speaking translators, and the original panel was contacted in case of any issues. Then, the Arabic and English versions of the questionnaire were validated by six different specialists (infectious diseases, public health, psychiatrists, family medicine, rehabilitation, and internal medicine) to confirm its comprehension and cultural acceptability.

#### 2.3.2 The second phase (the pilot phase)

*The second phase (the pilot phase) is* to assess the procedures and the pathway and to examine the data collection tool’s content validity, accuracy, and clarity of different items. It was conducted by two well-trained family physicians in December 2021 on 600 COVID-19 cases at least 4 weeks after the onset of infection using a validated, well-structured questionnaire.

The participants were asked to rate the questionnaire’s organization, clarity, and length, as well as provide a general opinion. Following that, certain questions were revised in light of their input. To check for reliability and reproducibility, the questionnaire was tested again on the same people 1 week later. We calculated a Cronbach’s alpha of 0.78 for the final questionnaire (as in Section 2.3.4). The final data analysis did not include the data collected during the pilot.

#### 2.3.3 The third phase (the pathway preparation phase)

**Figure 1:**
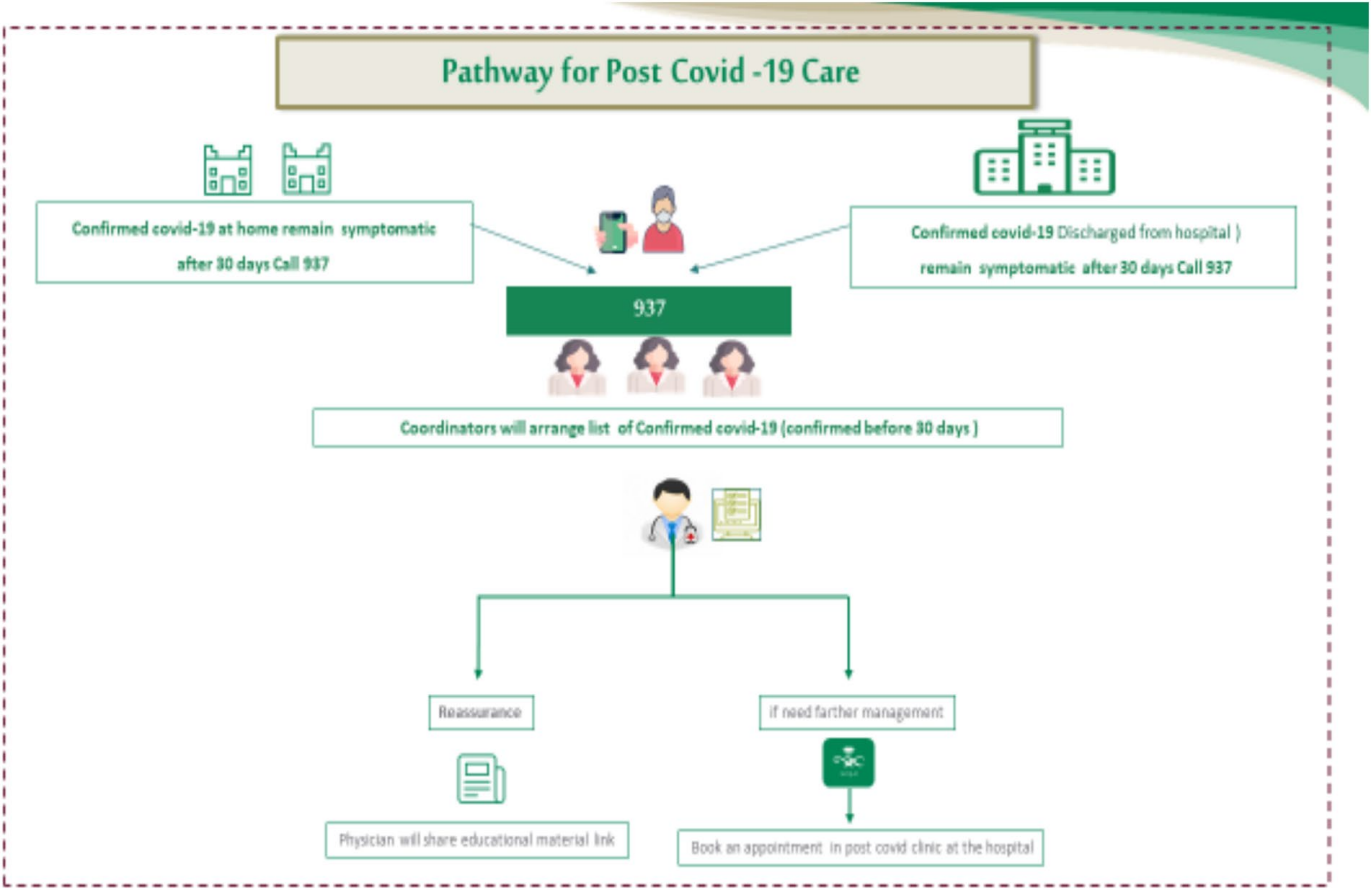
Pathway for post-COVID-19 clinical care in KSA.

The pathway phase preparation is based on available resources and pilot results, as illustrated in Figure 1.

#### 2.3.4 The virtual clinical evaluation phase (data collection tool and method)

A skilled family physician conducted the assessment or data collection through the Medical Consultation Centre (937) in SA, which offers telephone medical consultations and an e-health medical app (Anat). The average duration of the virtual consultation was 10 min, ranging from 8 to 20 min. The physician provides the service from 8 a.m. to 4 p.m. on all days of the week except weekends.

The structure of the assessment (data collection tool): After receiving permission to participate, participants provided informed consent. (1) The demographics, special habits, comorbidities, and medications of the patient population. (2) The history and symptoms of acute COVID-19 are classified as critical, severe (managed in the hospital), and not severe (managed at home), and the vaccination status. (3) The clinical assessment of PCCs by:

*A list of 50 PCC symptoms Following COVID-19*, a range of physical, social, and psychological effects are linked (15).

*The Medical Research Council (MRC) measured* the perceived impairment or disability related to breathing. The five statements that make up the MRC Dyspnea Breathlessness Scale questionnaire are rated from 1 to 5 to indicate 120 different levels of exertional dyspnea, ranging from mild to severe. Participants select the grade level that most closely matches their own. MRC grades 1 and 2 were used to classify mild dyspnea, grades 3 and 4 for moderate dyspnea, and grade 5 breathlessness for severe dyspnea. The two categories of MRC severity grouping are moderate (breathlessness when rushing on the legs and stopping for a breath after taking a few steps) and severe (walking slower than people of the same age and being too breathless to leave the house or breathe when dressing) (16).

*Questionnaire on Chronic Fatigue Syndrome (CFS)* (15): An anchored ordinal scale with the values 0 (no symptom), 1 (insignificant), 2 (mild), 3 (moderate), and 4 (severe) was used by the respondents to rate their level of fatigue as well as the eight auxiliary criteria during the preceding 6 months. Following the addition of the eight auxiliary criteria, the patients were categorized as follows: normal (a score of 14 for none, trivial, or mild fatigue), chronic idiopathic fatigue (CIF) (a score of 14 for moderate or severe fatigue), CFS-like with insufficient fatigue syndrome (a score of 14 for none, trivial, or mild fatigue), and CFS (a score of 14 for moderate or severe fatigue).

*The innovative Patient Self-Reported Functional Status (PCFS) scale* was used to assess and track the functional effects of PCCs. The PCFS scale, on average, includes all functional limitations for 1 week (17).

*The World Health Organization’s five wellbeing index (WHO-5)* was used to measure psychological wellbeing (19). Over the course of the preceding 2 weeks, participants were asked to score five positively worded statements on a Likert scale that went from always (five points) to never (zero points). Each component of the score is multiplied by four to arrive at a final score that ranges from 0 to 100.

*Depression screening* regarding the prevalence of low mood and anhedonia over the previous 2 weeks, according to the Patient Health Questionnaire (PHQ-2 scale), on the Likert depression scale, 0 meant not at all, a few, two meaning more than half the days, and three meaning almost every day. It ranged from 0 to 6 based on the total score (20).

*Generalized Anxiety Disorder-2 (GAD-2) Assessment:* This is a simple and fast way to do preliminary screening. For generalized anxiety disorder, a Likert scale with the following categories is used: not at all, a few days, more than half a day, and almost every day (22).

#### 2.3.5 The intervention phase (the management plans of PCCs)

Based on the physician’s virtual clinical assessment, as stated in the previous section, the recommended management plans based on the Saudi Guidelines for Post-COVID-19 Clinical Care (22) were either:

*Nothing*; *Reassurance:* Reassure them that support will continue to be provided as new information emerges; *Clinical Education:* Advise cases in which PCCs are not yet well understood and continue to discuss progress and challenges and reassess goals as necessary. Frequently, after COVID-19, symptoms were not explained by or out of proportion to objective findings and should not be dismissed, even if there is not yet a complete understanding of their etiology or severity; *Referral:* to a dedicated clinic in Ma’uad through an active referral system for post-COVID-19 care either to the nearest one from the 2,400 primary healthcare centers (PHCCs) or to post-COVID-19 clinics in hospitals all over the 20 health regions all over the SA.

*The indication of referral:* The presence of two or more of the following demographic variables in a symptomatic case: (I) Age more than 60 years, obesity = BMI of 30 or greater, number of comorbidities (more than three comorbidities); (II) Has one of the following: autoimmune disease, cancer, an on-dialysis history of cancer, or immunosuppression medication; (III) Requires clinical examination (specific organ or system lesion), e.g., the high Medical (MRC) dyspnea scale; (IV) Requires further investigation (laboratory, imaging, medications, or prescriptions); (V) Based on the clinical assessment: chronic fatigue syndrome (CFS) questionnaire (chronic idiopathic fatigue (CIF) with three symptoms, CFS-like with insufficient fatigue syndrome 1–2, and CFS with four symptoms) and World Health Organization five wellbeing index (WHO-5) with values from 0 to 50.

#### 2.3.6 The response rate of the referred cases after the virtual assessment by the family physicians

Through Ma’uad (an active referral system) for post-COVID-19 care, either to primary health care centers (PHCCs) the closest of the 2,400 PHCCs or to post-COVID-19 clinics in hospitals across the 20 health regions in SA (either attendance, no-show, or cancel the appointment).

### 2.4 Statistical analysis

The data collected were coded and examined using SPSS software, version 27. At baseline and at least 4 weeks’ post-infection, the frequency and proportions (%) of COVID-19 symptoms and indicators were determined. After the data in the sub-sample were cleaned, the baseline characteristics of the sample were compiled using descriptive statistics.

Returning to pre-COVID-19 health levels was the post-assessment referral’s binary outcome. Dependent variables included age, gender, hospitalization history, comorbidities, and the total number of symptoms of the participants. Then, to investigate the independent relationships between each putative factor and the desired outcome, we methodically created a final logistic regression model.

We first included all of the factors that were significant (*p* < 0.05) in the univariable analysis in the stepwise regression technique. Then, we kept the significant (*p* < 0.05) components of the final model and evaluated the potential significance of each non-significant variable. To ascertain each factor’s statistical significance, we employed likelihood ratio tests.

The variables that were kept in the model were participant age, gender, healthcare worker, comorbidities, number of symptoms and signs at the onset of infection, severity of COVID-19 infection, and interaction between the number of symptoms and hospital admission based on this iterative testing approach in stepwise regression. The adjusted odds ratios (OR, 95% confidence intervals) were computed and released.

## 3 Results

**Figure 2:**
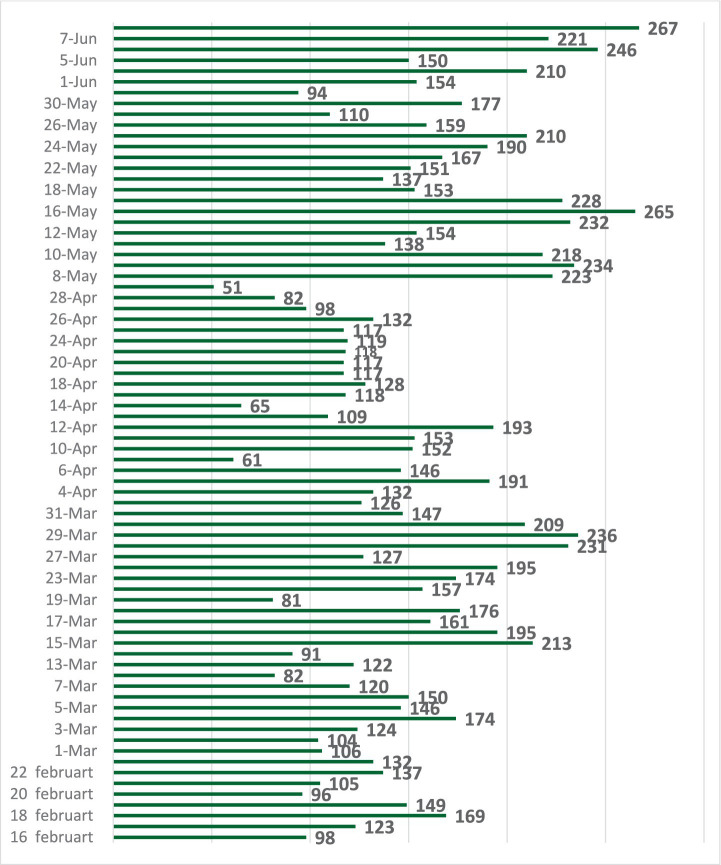
The daily number of calls by the family physicians in the medical consultation center.

**Figure 3:**
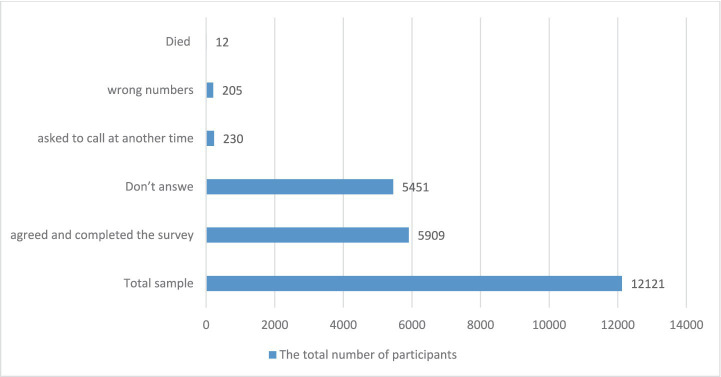
The response rate to virtual clinical assessment calls.

The response rate to the virtual assessment response: Across the 14 weeks of the study, the median number of calls per week was 177, which varies across different weeks and ranges from 98 to 276 calls per day (Figure 2). Out of the 12,125 registries that received the virtual assessment calls, only 5,913 (48.8%) agreed and completed the survey; 5,451 (45.1%) did not answer; 314 (2.6%) refused to complete the virtual assessment; 230 (1.9%) asked to call at another time; 205 (1.7%) were wrong numbers; and 12 (0.1%) died. Therefore, we limited the analyses to this final sample of 5,909 (48.8%) (Figure 3).

Concerning COVID-19 cases’ general and demographic characteristics (Table 1): Among the 5,913 participants who accepted the virtual assessment, the following demographics made up the majority: 74.5% were between the ages of 18 and 65; 54.6% were women; 89.3% were Saudis; 58.9% were married; 34.2% worked in public service; 51.2% held a bachelor’s degree; 49.3% had a normal BMI, but only 11.0% had a comorbidity; and 16.7% were regularly prescribed medication users.

**Table 1:** Demographic and general characteristics of the studied COVID-19 cases.

Variables	Total *N* = 5,913 F (%)
Age (y)	
Median (mean ± SD)	33(32.01 ± 19.8)
(Range)	(5–93)
Nationality	
Saudi	5,278(89.3)
Non Saudi	635(10.7)
Occupation	
Child	358(6.0)
Student	1,171(19.8)
Housewife/not working/retired	1,349(22.8)
Work in contact with the public	2024(34.2)
Do not work in contact with the public	755(12.8)
Sex	
Male	2,683(45.4)
Female	3,229(54.6)
Marital status	
Child	502(5.8)
Single	1790(30.3)
Married	3,484(58.9)
Divorced/widow	136(2.3)
Smoking	
Not-smokers	4,491(76.0)
Passive smokers	313(5.3)
Smokers	956(16.2)
Ex-smoker	152(2.6)
Level of education	
Child	285(4.8)
Illiterate	77(1.3)
Primary/read and write	748(12.6)
Preparatory	473(8.0)
Secondary	1,208(20.4)
University or higher	3,027(51.2)
Comorbidity	649(11.0)
BMI (kg/m^2^)	
Underweight	235(3.9)
Normal	2,915(49.3)
Overweight	935(15.8)
Obese	49(0.83)
Do not know/not sure	1779(30.1)
On regular medications	981(16.7)

The COVID-19 vaccine’s history and the acute symptoms of the virus (Table 2): Out of the total participants, 1,622 (57.5%) received three doses of the vaccination, while 716 (12.2%) did not receive any vaccination; 5,352 (90.6%) of the participants had symptoms in the majority of cases. Fever (4,118, 69.7%), cough (2,225, 38.2%), sore throat (1,674, 28.3%), headache (1,431, 24.2%), nasal congestion, and myalgia [1238] (20.9%) were the most frequently reported acute COVID-19 symptoms. Merely 63 (1.1%) out of those who reported symptoms required additional treatment (re-hospitalization, follow-up plan, or referral to PHCCs), while 5,813 (98.4%) had complications and were treated at home.

**Table 2:** The history of COVID-19 vaccination and SARS-CoV2 infection of the participants.

Variables	Total (*N* = 5,913) F (%)
COVID-19 vaccination	
Unvaccinated	718(12.1)
Single dose	114(1.9)
Two doses	620(10.5)
Three doses	1,624(57.5)
Four doses	17(0.3)
The acute COVID-19 symptoms	
Asymptomatic	560(9.4)
Symptomatic	5,358(90.6)
The median number of PCC symptoms (IQR)	
Median (mean ± SD) (range)	7(3–10)
The self-reported symptoms of acute COVID-19 symptoms^#^	
Fever	**4,118(69.7)**
Nasal congestion	**1,327(22.5)**
Fatigue	766(12.9)
Sore throat	**1,674(28.3)**
Nausea and vomiting	182(3.1)
dyspnea	704(11.9)
Cough	**2,257(38.2)**
Myalgia	**1,238(20.9)**
Dizziness	174(2.9)
Diarrhea	139(2.4)
Loss of appetite	44(0.7)
Loss of smell	313(5.3)
Loss of taste	413(6.9)
Headache	**1,431(24.2)**
Stomachache	65(1.1)
Chest pain	109(1.8)
Night sweats	18(0.3)
Conjunctivitis	16(0.3)
Blurring of vision	5(0.08)
Poor concentration	7(0.12)
Others	44(7.4)
Complications	
No	5,639(95.4)
Pulmonary complications	4(0.06)
Cardiac complications	3(0.06)
Neurological	3(0.06)
Psychiatric complications	1 (0.02)
Others	261(4.5)
Severity of SARS-CoV-2 infection	
Critical	24(0.4)
Sever (managed at hospital)	73(1.2)
Not-Sever (managed at home)	5,815(98.4)
Post-recovery management	
Nothing	5,846(98.9)
Re-Hospitalized	18(0.3)
Follow up plan.	42(0.7)
Referred to PHCCs	6(0.08)

^#^Multiple answers were allowed. Bold indicates the highest frequency.

The symptoms that PCCs self-reported, listed in Table 3: While 5,301 (89.7%) of the cases stated that their health had returned to before the infection, there was a notable decrease in the median number of symptoms from seven (IQR 3–10) at the beginning of the disease to three (IQR 1–6) after 4 weeks (*p* = 0.001), showing a significant and somewhat positive correlation (*r* = 0.61, *p* = 0.001) between the two. The majority of participants (4,973, 84.2%) did not report any PCCs, and the most common symptoms were fatigue (201, 3.4%), cough (246, 4.2%), dyspnea (209, 3.6%), loss of appetite or weight loss (43, 7.3%), and poor concentration (50, 8.4%).

**Table 3:** The self-reported post-COVID-19 symptoms among the participants.

Variables	Total *N* = 5,913 F (%)
The self-reported post-COVID-19 symptoms^#^	
Nothing	4,973(84.2)
Cough	**246(4.2)**
Dizziness	26(0.4)
Dyspnea	**209(3.6)**
Loss of appetite/loss of weight	**43(7.3)**
Nausea/vomiting	16(0.3)
Pain	147(2.5)
Chest pain	34(0.6)
Stomachache	101(1.7)
Joint pain	14(0.2)
Fatigue	**201(3.4)**
Sleep disturbances	106(1.9)
Diarrhea	12(0.2)
Loss of smell	80(1.3)
Loss of taste	0(0.0)
Loss of hearing/tetanus	9(0.2)
Headache	81(1.3)
Recurrent fever	12(0.2)
Memory impairment	134(2.3)
Poor concentration	**50(8.4)**
Psychiatric impairment	103(1.7)
Hair loss	67(1.13)
Skin rash	7(0.12)
Menstrual disturbances	47(0.7)
Others	57(0.9)
**The median number of PCC symptoms (IQR)**	3(1–6)
Return to the pre-infection health status	
Resolved	5,303(89.7)
Unresolved	610(10.3)
Duration to return to the pre-infection health status (d)	
Median (mean ± SD)	6 (6.3 ± 10.2)

^#^Multiple answers were allowed. Bold indicates the highest frequency.

The PCCs’ clinical assessment (Table 4): The majority of cases reported the following at least 4 weeks after the infection began: There were no functional status limitations in PCFS (5,596, or 94.7%), mild dyspnea in MRC (5,822, or 98.5%), moderate to severe dyspnea in 86 (1.5%), and normal chronic fatigue in PCFS (5,234, or 88.6%); a syndrome resembling chronic tiredness was present in 306 (5.2%), and a diagnosis of chronic fatigue syndrome was made in 68 (4.5%) cases. Only 821 people, or 13.9%, reported having good mental wellbeing in 1981, while 63 people (1.1%) reported having an abnormal PHQ-2 depression scale and 52 people (0.8%) reported having an abnormal GAD-2 anxiety scale.

**Table 4:** Post-COVID-19 clinical assessment among the studied group.

Variables	Total (*N* = 5,913) F (%)
Patient self-report functional status (PCFS) scale	
No functional status limits	5,597(94.7)
Mild restriction	154(2.6)
Moderate restriction	142(2.4)
Severe restriction	17(0.3)
Medical research council (MRC) dyspnea scale	
Mild dyspnea	5,824(98.5)
Moderate dyspnea	77(1.3)
Severe dyspnea	11(0.2)
None	1(0.00)
Anxiety (GAD-2)	
Median (mean ± SD) (range)	0 (0.11 ± 0.61) (0–6)
Normal (<4)	5,859(99.2)
Abnormal (4 or more)	54 (0.8%)
WHO—5 wellbeing score	
Mean ± SD (range)	71.4 0.3.1 ± 3.8 (0–100)
Less than 50 (poor wellbeing)	1982(33.5)
50 or <75	3,108(52.6)
75 or more	823(13.9)
Depression (PHQ-2)	
Median (mean ± SD) (range)	0(0.14 ± 0.66) (0–6)
Abnormal (4 or more)	65 (1.1)
Normal (<4)	5,848 (98.9)
Chronic fatigability	
No/unapplicable	5,044(85.3)
Short-term memory impairment	405(6.8)
Sore throat	117(1.98)
Lymph node	46(0.8)
Muscle pain	315(5.3)
Difficult sleep	285(4.8)
Extreme fatigability	389(6.7)
Joint pain	329(5.6)
Chronic fatigability	
Median (mean ± SD) (range)	1(1.5 ± 1.9) (1–22)
Normal	5,235 (88.6)
Syndrome resembling chronic tiredness	307(5.2)
Idiopathic chronic fatigue	102(1.7)
Chronic fatigue syndrome	269(4.5)

**Figure 4:**
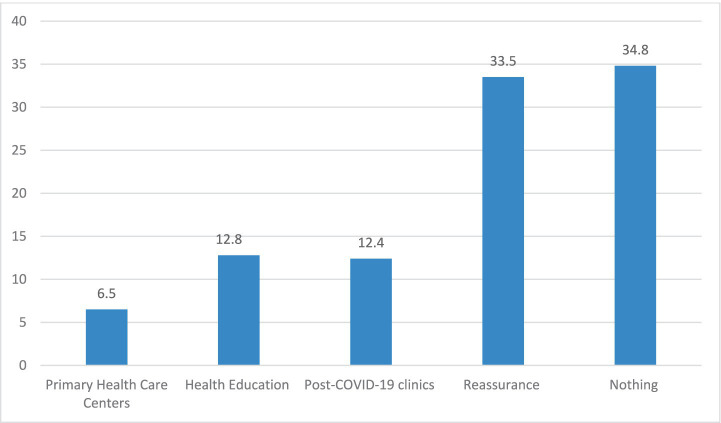
The required management.

Clinical management following COVID-19 (Figure 4): The skilled family physician completed a virtual clinical assessment of the cases and then provided the necessary management plans: of the recruited participants, 2056 (34.8%) required no referral; 1979 (33.5%) required reassurance; 12.8% required health education; 384 (6.5%) required referral to PHCCs; and only 12.4% required referral to the hospital’s post-COVID-19 clinics.

**Figure 5:**
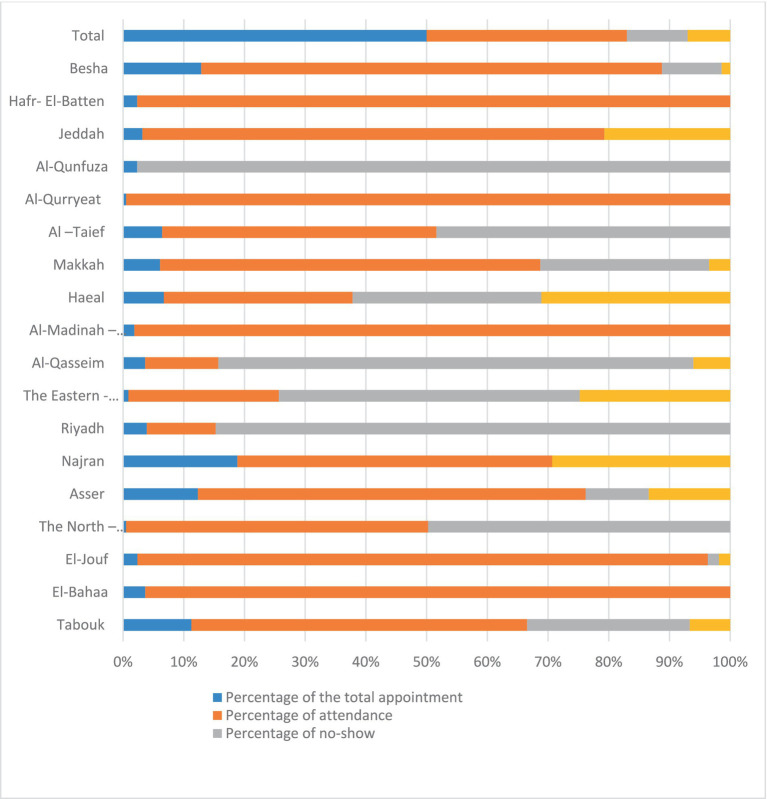
The total number of referral appointments, the attendance, the no-shows, and the canceled ones across the 20 health regions in SA.

Of the 418 appointments, 274 (66%) were attended, while 20% of the total were no-shows and 14% canceled their appointments; the percentages for no-shows and cancellations ranged from 0% to 35 (36.1%) (Figure 5).

As shown in Table 5, age, sex, vaccination status, BMI, and comorbidities were significant predictors of return to baseline health status according to the results of the univariate and multivariate analyses. Following completion of the virtual assessment status, the participant’s referral to post-COVID-19 clinical care at PHCCs or hospitals was significantly influenced by age, vaccination status, and comorbidities, as summarized in Table 6.

**Table 5:** Factors significantly associated with the participants’ reports of delayed return to the pre-COVID-19 baseline health status.

Variables	Univariable analyses		Multiple logistic regression model (adjusted ORs)	
Demographics	OR (95% CI)	*P*	OR (95% CI)	*P*
Age (groups)				
Adult (18 y– < 65 y)	Reference	–	–	–
<18 y	0.50 (0.34–074)	0.025*	0.0.74 (0.62–0.88)	0.020*
65 y or more	12.68 (1.71–4.20)	<0.001*	12.63 (1.64–4.23)	<0.001*
Sex	Reference			
Female		–	–	–
Male	0.55 (0.48–0.61)	<0.001*	0.72 (0.62–0.82)	<0.001*
Severity of SARS-CoV-2 infection	4.83 (3.72–6.97)	0.023*	1.93 (1.78–5.10)	0.372
Smoking status				
Never smoked	Reference	–	–	–
Previous smoker	1.22 (1.00–1.49)	0.050	–	–
Current smoker	1.23 (1.05–1.45)	0.012*	–	–
Unknown	1.01 (0.60–1.69)	0.975	–	–
Vaccination status				
Unvaccinated	3.7 (1.34–6.74)	<0.001*	1.75 (1.48–2.16)	0.001*
Single dose	0.84 (0.77–0.91)	<0.001*	0.60 (0.46–0.77)	<0.001
Two doses	0.27 (0.08–0.94)	0.040*	0.47 (0.12–1.81)	0.274
Three doses	References		–	–
BMI**/**overweight and obese	1.97 (0.99–3.11)	<0.001*	1.91 (1.00–2.2)	<0.001*
Comorbidities	3.7(1.9–11.7)	<0.001*	2.1(1.0–3.6)	<0.001*

*p < 0.05, there was a statistically significant difference.

**Table 6:** Factors significantly associated with the participant’s referral to Post-COVID-19 Clinical Care at PHCCs or hospitals after completing the virtual assessment.

Variables	Univariable analyses		Multiple logistic regression model (adjusted ORs)	
Demographics	OR (95% CI)	*P*	OR (95% CI)	*P*
Age (groups)				
Adult (18– < 65 y)	Reference	–	–	–
<18 y	0.22 (0.14–0.35)	0.00*	0.74 (0.62–0.88)	0.020*
65 y or more	2.03 (1.64–4.23)	<0.001*	1.28 (0.91–1.82)	0.16
Sex-Female	0.55 (0.48–0.61)	<0.001*	0.72 (0.62–0.82)	<0.001*
Severity of SARS-CoV-2 infection	13.83 (6.72–18.97)	<0.001*	2.93 (1.78–5.10)	0.372
Vaccination status				
Unvaccinated	1.3 (1.34–6.74)	<0.001*	1.05 (0.48–1.16)	0.001*
Single dose	0.516 (0.360–0.74)	<0.001*	0.60 (0.46–0.77)	<0.001*
Two doses	0.59 (0.50–0.69)	<0.001*	0.81 (0.61–1.08)	0.159
Three doses	References	–	–	–
BMI/overweight and obese	1.33 (0.65–2.7)	0.06		
Comorbidities	1.8 (1.182–3.0)	0.008*	1.34(1.02–2.1)	0.03*

*p < 0.05, there was a statistically significant difference.

## 4 Discussion

This is a novel national project that was carried out in Saudi Arabia using digital transformation technology (Anaa, Ma’uad, and virtual clinics at the medical consultation center) by well-trained family physicians to virtually assess the PCCs among all COVID-19 cases after 1 month from the infection and provide the required management based on the well-structured published Saudi Guideline on Post-COVID-19 Clinical Care. This study provides important and generalizable evidence.

### 4.1 The response rate to the virtual clinical assessment calls

Out of the 12,125 patients who received virtual assessment calls in SA, 5913 (48.8%) participants accepted and completed the evaluation. This may be due to the residents’ trust in the telehealth services, which began operations in SA in early 2017, and the fact that they were enthusiastically embraced and utilized as a crucial component of the Vision 2030 strategy for enhancing healthcare, especially during the pandemic (23), while only 230 (1.7%) declined, especially when asked about their marital status as a result of cultural factors in the SA.

Although the RR increased across the study weeks as a result of the nationwide media campaign, nearly half of the target population (5,451, or 45.1%) did not respond, and 314, or 2.6%, requested a callback because the service was available from 8 a.m. to 4 p.m. with a single call. Despite the fact that the service was provided from an official 937 number, it is believed to be one of the most significant obstacles or restrictions to the virtual services offered.

### 4.2 The frequency of the self-reported symptoms of acute SARS-CoV-2 infection and PCCs

Our key finding was that the self-reported PCCS prevalence was 936 (15.8%). This frequency varies from country to country; for instance, it is lower than the 79.4% reported in SA (December 2020) at least 4 weeks after the infection (18), in the United States, higher than what was reported in October 2022, when 6.9% of adults ever had PCCs and 3.4% of adults currently had PCCs at least 3 months after the infection (24), while in a meta-analysis using 194 studies including 735,006 participants (July 2022), 54% of COVID-19 survivors experienced at least one unresolved symptom regardless of hospitalization status 4 months after the infection (25).

The various figures provided by the aforementioned studies are likely to reflect differences in response rates, the self-reporting, PCCs criteria, the age and baseline clinical conditions of the participants, the assessed symptoms, PCCs detection techniques, and the different strains of the virus. Consequently, the decreased frequency of the PCCs in this study may be attributed to the shorter follow-up duration (4 weeks after the infection) and the children’s self-reported PCCs with parental consent. Moreover, 14% of the participants were less than 16 years old, and verbal children are less likely to report specific symptoms in their younger years, which introduces a variable risk of bias as a result (26–29).

Poor concentration (50, 8.4%), loss of appetite or weight loss (43, 7.3%), coughing (246, 4.2%), dyspnea (209, 3.6%), and fatigue (201, 3.6%) were the most prevalent symptoms. In contrast to the results of earlier regional studies, fatigue (45.2%) and muscle aches (38.2%) were the most common complaints (18). An earlier meta-analysis found that fatigue was the most prevalent symptom at all times up to more than a year after the onset of COVID-19 disease in over 30% of hospitalized patients, those with a longer follow-up of 4 months, and in international settings. At 3–6, 6–9, 9–12, and >12 months, 32, 36, 37, and 41%, respectively, of 257,348 patients from 63 studies with at least 12 weeks of follow-up had fatigue (29).

Among other significant findings, 23.9% of participants reported shortness of breath, and 30.5% of individuals reported joint problems. In addition to headaches, dizziness, loss of taste and smell, sleeplessness, appetite loss, and difficulty concentrating, more than 20% of patients have also reported experiencing additional symptoms, such as nausea, vomiting, and fatigue. The persistence of numerous symptoms in our patients is comparable to what Davis et al. (30) discovered in an international cohort study, where 66 symptoms were monitored for 7 months after the onset of COVID-19 disease. A recent meta-analysis of 15 trials involving 47,910 patients included documentation of 50 long-term symptoms of COVID-19 illness, the most common of which were fatigue (58%), headaches (44%), attention disorders (27%), and hair loss (25%) (15).

In addition to the high vaccination coverage in SA, 1622 (57.5%) received three doses of the COVID-19 vaccine, only 716 (12.1%) did not, and vaccinations were a significant protective risk factor for rapid recovery to pre-infection health status (OR = 0.84, 95% CI = 0.77–0.91) and for referral to post-COVID-19 (CI 0.36–0.74); thus, the variation in frequency of the common presenting symptoms of acute SARS-CoV-2 infection and PCCs may be due to this. Moreover, the numerous viral strains undergo constant mutational change (as in January, the main strain in SA was the delta strain); depending on past infection history and other factors, symptoms may be present or more severe (5, 26).

There is a moderately significant correlation between the number of self-reported SARS-CoV-2 symptoms and the number of PCCs. However, it aligns with research that found adults who had five or more symptoms during an acute illness were more likely to have PCCs (31, 32).

### 4.3 Post-COVID-19 clinical evaluation

Although only 936 (15.8%) of cases self-reported PCCs, by professional clinical assessment, 1933 people (33.5%) got a wellbeing score below 50, which means they were poor. This was attributed to the fact that a professional clinical assessment showed that 11.4% of participants had abnormal CFS and 5.5% had functional limits. In accordance with the findings, 10 participants (0.2%) had severe dyspnea, 5,822 (98.5%) had mild dyspnea, 76 (1.3%) had moderate dyspnea, 52 (0.8%) had anxiety disorder, and 63 (1.1%) had depression disorder.

In agreement with prior research, we found that professional evaluation and self-rating yield distinct results. These differences may be attributable to the personalities and demographics of the patients; as a result, it is crucial to accurately assess the health status of patients to prevent the wastage of scarce health resources and enable patients to receive care via less arduous means. On the contrary, it could reduce medical personnel’s workload. Professional evaluation is still the gold standard for diagnosing health disorders; it involves a thorough assessment of the patient’s symptoms by clinically trained, experienced professionals (33–35).

Viruses in general, and SARS-CoV-2 especially, have been linked to CFS; therefore, we used an 8-item CFS questionnaire to find out what kind of exhaustion the PCCs were experiencing. Abnormal CFS was found in 11.4% of them, which is much lower than the 38.4% frequency reported in other studies for at least 4 weeks. In contrast, at least one COVID-19 study reported 25 of 29 CFS symptoms in a recent meta-analysis of CFS symptoms in COVID-19 disease, comprising 21 studies. In addition, subsequent to the recovery from SARS, findings comparable to ours have been published. According to the report by Lam et al., 27.1% of recovered SARS patients met the criteria for CFS (36–39).

### 4.4 Predictors for return to the pre-infection health status, or referral to PHCCs, or post-COVID-19 clinics in hospitals

After univariate and multivariate analyses, several factors were found to predict the delayed return to pre-illness status. Female gender was associated with an odds ratio (OR) of 0.72 [95% confidence interval (CI): 0.63–0.82, *p* < 0.001]. Numerous studies (22, 25, 38, 39) have unanimously confirmed this observation. The consistent observation of a higher risk of developing post-COVID-19 syndrome in females is not completely understood; however, it may be partially attributable to the well-known sex dimorphism observed in certain disorders, such as autoimmune diseases (28).

There was a consistent decrease in the likelihood of returning to the pre-infection health status and an increase in the likelihood of referral after the virtual assessment. Being 54 years old had the highest OR (OR 2.60, 95% CI 1.59–4.25, *p* = 0.001). This is because the risk of severe COVID-19 infection increases with age. People are susceptible to developing COVID-19 with rapidly deteriorating symptoms because of their weakened immune systems and other health issues. Therefore, they are more susceptible to cytokine storms caused by viruses. This can affect multiple systems and result in potentially fatal respiratory failure (28). As a result, it necessitated close monitoring and additional clinical and laboratory investigations.

Failure to return to the baseline state before the illness was also linked to hospitalization (OR 0.35, CI 0.21–0.59, *p* < 0.001), the number of symptoms (OR 0.91, CI 0.89–0.91, *p* < 0.001), and a number of other conditions, such as asthma and arrhythmias. These results once again concur with those of other studies (37–39). This finding is consistent with all other research, including an earlier conclusion from a smaller cohort of hospitalized COVID-19 patients. The length of hospital stays, the number of symptoms, and advanced age were risk factors associated with PCCs (40). Nonetheless, future research is required on this topic.

### 4.5 Post-COVID-19 clinical management

The overall rate of referrals from virtual post-COVID-19 clinics to PHCCs, or post-COVID-19 clinics in hospitals, was 6.5%, which is higher than the average referral rate for telephone consultations in SA, which accounts for approximately 2% of primary healthcare center visits (41). The majority of participants were unaware of the PCCs and their health needs for further investigations.

Of the 418 Ma’uad referral appointments made to PHHCs or post-COVID-19 clinics in hospitals following the virtual assessment, 144 (34.5%) PCCS did not attend their appointments [87 (20%) had not shown for their appointment and 57 (14%) canceled their appointment]. Numerous factors may be to blame for this, including the participants’ perception that these services are proactive and primitive and that they do not need them; time; cost; demographics; accessibility factors such as location, waiting times, and working hours; their varying degree of satisfaction with the current capacity of PHCCs; the services offered; and the availability of health specialists in PHCCs (42–44).

Thus, no-show is still a critical challenge worldwide, including the SA problem, which has a significant impact on revenues, costs, and the use of resources, despite the development of medical appointment systems in SA by sending reminder messages two times before the date to decrease the number of people not attending the appointment (43). While in various healthcare settings, no-show rates range from 12 to 80% (45–47).

Across the 20 health regions, the percentage of appointments that are canceled or not shown up varies from 0 to 35%, or 0 to 100%, respectively. For example, in Al-Qurryeat, Al-Madinah-El-Monawarrah, El-Bahaa, and Hafr-El-Batten, 100% of the scheduled appointments were attended. This can be explained by many cultural, traditional, and adaptations to using the cancelation services. Moreover, nearly 56% of PHCCs are in rural areas. Similarly, rural areas have higher densities of PHCCs than urban areas, which can be explained by the very small size of rural populations in Saudi Arabia (only 17% of the population lives in rural areas) (48, 49).

### 4.6 Limitations

Our study has some limitations. First, being a call-virtual assessment, the high percentage of low response rates may affect the results. Second, COVID-19 cases from an earlier COVID-19 epidemic wave were not included in our research. Third, the lack of knowledge regarding the long-term effects of COVID-19 at this time may have had an impact on the reporting of relevant PCCs. Finally, because we did not conduct a pre-COVID-19 baseline health evaluation, it is impossible to differentiate between pre-existing issues and those associated with the COVID-19 condition. In spite of these limitations, evidence from other international studies supports our conclusion.

### 4.7 Strengths

This study’s strengths is that: (1) It examines how the national virtual services (Anat, Ma’uad, and virtual clinics at the medical consultation center) can provide effective health services; (2) This kind of level and depth of snapshot is essential in exploring the current state, gaps, and barriers across the nation and is crucial for informing future steps that will refine and improve the healthcare system; (3) This study provides a comprehensive and well-defined approach to addressing all PCCS-related issues in terms of symptoms, management, and attitude in a large number of cases of all age groups; and (4) Ensuring the integration between the various health sectors (public health agencies, digital transformation technology, hospital administrative services, and the public) that offer primary care services and bringing the decision-making process together.

## 5 Recommendation

(1) We recommended alerting COVID-19 outpatients to possible COVID-19 long-term effects. (2) To better manage and support patients who are at higher risk for persistent symptoms, doctors should be aware of the various causes of symptoms, such as fatigue, cognitive and neurologic symptoms, and dyspnea. They should also seek a differential diagnosis to avoid misinterpretation. (3) The creation of an outpatient clinical environment to assess, track, assist, and oversee recovered cases following COVID-19. (4) Creating a well-organized post-COVID-19 management protocol and guidelines based on national evidence-based findings. (5) To close any gaps or omit any information about the clinics or procedures, more research is required. Long-term research is also necessary to determine the etiology of the disease and show how effective the treatments are. Therefore, we advise carrying out more research and setting up plans to look into the underlying cause and create a workable strategy to deal with this problem. (6) Almost one-third of the cases that were referred either canceled or did not show up for their Ma’uad appointment, and more than half of the calls went unanswered. We advise carrying out more studies, setting up plans to look into the root cause, and creating a workable strategy to deal with this problem. (7) Its critical to reassess the necessary services to ensure that they are tailored to each region’s needs. It is important to consider the distribution, culture, and usage of digital technology services.

## 6 Conclusion

In SA, the response rate for the usage of digital health technology, including Anaa, Ma’uad, and virtual remote clinics at the medical consultation center, in the detection and management of PCCs was low, and no-show was the main limitation or challenge. PCCs’ professional evaluation reveals distinct PCCs compared to self-reporting PCCs. Therefore, the professional assessment of the health status of PCCs is critical to receive optimal care.

Although PCCs are relatively common health conditions, the majority require nothing more than reassurance and health education. The severity of the SARS-CoV-2 infection, age group, sex, vaccination status, and body mass index were significant predictors of returning to the health level before the infection, while age, vaccination status, and comorbidities were factors significantly associated with the participant’s referral to post-COVID-19 clinical care at PHCCs or hospitals.

## Data Availability

The raw data supporting the conclusions of this article will be made available by the authors, without undue reservation.
