# Correction: Virtual post-COVID-19 clinics in Saudi Arabia: navigating the effect of the pandemic with a national project

**DOI:** 10.3389/fpubh.2025.1709022

**Published:** 2025-10-23

**Authors:** Faisal Alenezi, Aeshah Alsagheir, Samar Amer, Lamyia Alzubaidi, Abdul-Aziz S. Alhomod, Tareef Alamaa

**Affiliations:** ^1^Deputy Minister Assistant for Hospitals, Services, Ministry of Health, Riyadh, Saudi Arabia; ^2^Family and Home Health Care Consultant, Assistant Agency for Hospitals Services, Ministry of Health, Riyadh, Saudi Arabia; ^3^Department of Public Health and Community Medicine, Zagazig University, Zagazig, Egypt; ^4^Assistant Agency of Public Health, Ministry of Health, Riyadh, Saudi Arabia; ^5^Consultant Diabetologist, Director of Technical Affairs and Ada'a in the Deputy for Hospitals Services, Ministry of Health, Riyadh, Saudi Arabia; ^6^Department of Emergency Medicine, King Fahad City, Riyadh, Saudi Arabia; ^7^Innovation and Regulatory Sandbox, Seha Virtual Hospital, Riyadh, Saudi Arabia; ^8^Deputy Minister of Curative Services, Ministry of Health, Riyadh, Saudi Arabia

**Keywords:** post-COVID-19 conditions, virtual clinics, digital transformation, Saudi Arabia, assessment, COVID-19, medical appointments, SARS-CoV-2

There was a mistake in the Ethics statement that was erroneously given as “Ethical approval was granted by the Prince Nourah University (PNU) Institutional Review on March 29, 2021, of the Exempt Type IRB Registration Number with KACST, KSA for the study involving humans in accordance with the local legislation and institutional requirements. Participants provided with informed consent before participating in the virtual assessment calls. The virtual assessment calls did not include sensitive or private questions”.

The correct Ethics statement is “The Institutional Review Board of the Ministry of Health, Saudi Arabia, waived the requirement for ethical approval for this public health service project, which was implemented free of charge by the Ministry of Health's virtual consultation call center for all registered COVID-19 cases in the national HESN registry system, with the endorsement of all relevant stakeholders within the Ministry of Health. Informed verbal consent was obtained from all participants during the virtual consultation calls. This waiver was granted in accordance with the national ethical framework, the HESN declaration, and in line with the principles of the Bahrain Declaration, which recognize that certain public health surveillance and service delivery projects may not require additional institutional review board approval.”

The original version of this article has been updated.

